# Reactive Oxygen Species: Do They Play a Role in Adaptive Immunity?

**DOI:** 10.3389/fimmu.2021.755856

**Published:** 2021-11-22

**Authors:** Esen Yonca Bassoy, Michael Walch, Denis Martinvalet

**Affiliations:** ^1^ International Society of Liver Surgeons (ISLS), Cankaya Ankara, Turkey; ^2^ Departments of Immunology and Cancer Biology, College of Medicine and Science, Mayo Clinic, Scottsdale, AZ, United States; ^3^ Faculty of Science and Medicine, Department of Oncology, Microbiology and Immunology, Anatomy Unit, University of Fribourg, Fribourg, Switzerland; ^4^ Department of Biomedical Sciences, University of Padua, Padova, Italy; ^5^ Veneto Institute of Molecular Medicine, Padova, Italy

**Keywords:** reactive oxygen species, adaptive immunity, T lymphocytes, B lymphocytes, tumor microenvironment

## Abstract

The immune system protects the host from a plethora of microorganisms and toxins through its unique ability to distinguish self from non-self. To perform this delicate but essential task, the immune system relies on two lines of defense. The innate immune system, which is by nature fast acting, represents the first line of defense. It involves anatomical barriers, physiological factors as well as a subset of haematopoietically-derived cells generically call leukocytes. Activation of the innate immune response leads to a state of inflammation that serves to both warn about and combat the ongoing infection and delivers the antigenic information of the invading pathogens to initiate the slower but highly potent and specific second line of defense, the adaptive immune system. The adaptive immune response calls on T lymphocytes as well as the B lymphocytes essential for the elimination of pathogens and the establishment of the immunological memory. Reactive oxygen species (ROS) have been implicated in many aspects of the immune responses to pathogens, mostly in innate immune functions, such as the respiratory burst and inflammasome activation. Here in this mini review, we focus on the role of ROS in adaptive immunity. We examine how ROS contribute to T-cell biology and discuss whether this activity can be extrapolated to B cells.

## Introduction

Reactive oxygen species (ROS) include both radical and non-radical species and are formed by the partial reduction of oxygen. The radical species, e.g., superoxide anion (O^2-.^), hydroxyl radical (.OH), and nitric oxide (NO), have unpaired electrons (1, 2). In contrast, the non-radical products, e.g., hydrogen peroxide (H_2_O_2_), hypochlorous acid (HOCl), and peroxynitrite (ONOO^-^), do not have unpaired electrons but remain powerful oxidizing agents (1). Interestingly, cellular enzymatic systems such as the nicotinamide adenine dinucleotide phosphate hydrogen (NADPH) oxidases, the myeloperoxidases, the nitric oxide synthases (NOS), the monooxygenase activity of cytochrome P450, xanthine oxidase, monoamine oxidase (MAO) and the mitochondrial respiratory chain are sources of the primary radical species (O^2-.^, NO, and H_2_O_2_) (1, 3). At low concentrations, which can be handled by the cellular antioxidant system, O^2-.^, NO and H_2_O_2_ are necessary for signal transduction, cell migration, cell differentiation, cell proliferation, vasoconstriction, inflammation, senescence and aging (4–14). This can be explained, in part, by the fact that to some extent primary species reactions with biomolecules are reversible and they are easily controlled by enzymatic and non-enzymatic antioxidant molecules of the cell antioxidant machinery (15–18).

Interestingly, although even at high concentrations O^2-.^, NO and H_2_O_2_ are not directly damaging to cells, they react with themselves or with metal ions to produce the extremely toxic secondary reactive species.OH, ONOO^-^ and HOCl. These secondary species are poorly controlled and rapidly and irreversibly react with virtually all classes of biomolecules causing oxidative damage. The accumulation of ROS can lead to a state of oxidative stress when the endogenous antioxidant machinery of the cell is overwhelmed (19–24). Consequently, the cells accumulate oxidative damage within the DNA, lipids and proteins, causing cellular dysfunction and cell death (19–23). Excessive ROS production plays a major role in the initiation and amplification of cell death by modulating many signaling pathways. Consequently, ROS levels are contributing determinants for various forms of cell death, including apoptosis, necrosis/necroptosis, ferroptosis, pyroptosis and autophagic cell death (25–32).

The immune system has the unique ability to distinguish self from non-self to protect the host organisms from a plethora of microorganisms and toxins (33–35). It eliminates foreign entities (pathogens and toxins) but tolerates the self (host’s own tissues) and its associated microbiota (33, 36, 37). The innate immune system, the components of which are already present before any pathogenic intrusion, is fast acting. It relies on anatomical barriers (the skin and the mucosa lining the respiratory, gastrointestinal and urogenital tracts) to prevent foreign entities from entering the organism (33, 34). These anatomical barriers are reinforced by soluble factors (complement system, pentraxins, collectins and the defensins antimicrobial peptides) as well as by leukocytes (macrophages, dendritic cells, mast cells, neutrophils, eosinophils, natural killer [NK] cells) that neutralize pathogens or kill the infected cells (33, 34). The innate immune system is activated by the recognition of antigenic determinants common to a wide spectrum of microbes (the pathogen associated molecular patterns [PAMP]) and leads to a state of inflammation to alert and combat the ongoing infection (33, 34, 38). Importantly, the activated innate immune system delivers the antigenic information of the invading pathogens to activate the slower but highly potent and specific second line of defense known as the adaptive immune system. The adaptive immune response calls on T lymphocytes and B lymphocytes as, respectively, the effectors of the cellular adaptive immune response and as the antibody-producing cells with the essential functions of eliminating pathogens and establishing immunological memory (35, 39, 40).

ROS have been implicated in many aspects of the immune response to pathogens mainly related to innate immunity. Indeed, they have been proposed to be the common determinant of inflammasome activation, which is critical in the inflammatory process and thus necessary for an efficient immune response. ROS are also essential for pathogen killing by phagocytic cells, as illustrated in chronic granulomatous disease (CGD), an inherited disorder of NADPH oxidase characterized by recurrent and severe bacterial and fungal infections as phagocytes from these patients cannot do the respiratory burst. Here in this mini review, we focus on the role of ROS in adaptive immunity. We examine how ROS contribute to T-cell biology and briefly discuss whether these activities can be extrapolated to B cells.

## ROS and Lymphocyte Activation

**Figure 1:**
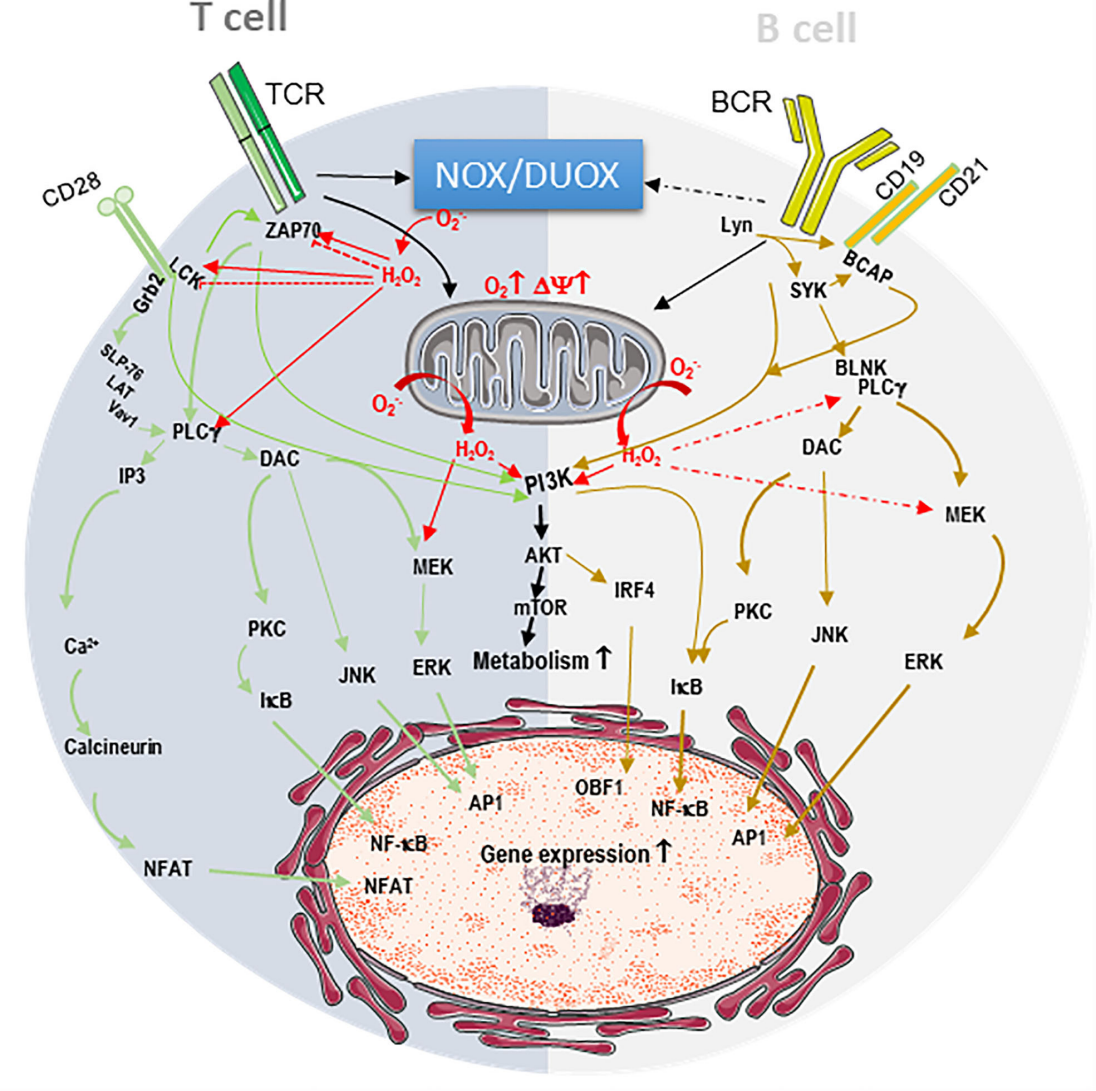
Endogenous ROS contribute to T and B cell receptor signaling. The engagement of the B-cell receptor (BCR) or T-cell receptor (TCR) and their respective co-receptors CD28 and CD19/CD21 triggers intracellular phosphorylation signaling cascades resulting in the activation of transcription factors AP1, NF-κB, NFAT, OCA-B/OBF-1 IRF-4, which are critical for T and B lymphocyte activation. In T cell, dark grey, TCR/CD28 stimulation induces the phosphorylation and activation of the kinases p56lck (LCK) and ZAP-70, the phospholipase Cg (PLCg). CD28 recruits growth factor receptor bound protein 2 (Grb2), which docks the complex formed by SH2 domain containing leukocyte protein of 76kDa (SLP-76)/Linker for activation of T-cells (LAT)/signal transducer protein Vav1. The latter complex recruitment closer to the membrane facilitating its activation by ZAP70. PLCg generates inositol 3 phosphate (IP3) to mobilize intracellular Ca2^+^ stores resultingin the activation of the phosphatase calcineurin. PLCg also generates diacylglycerol (DAC) to activate protein kinase C (PKC), c-Jun N-terminal Kinase (JNK) and mitogen-activated protein kinase/extracellular signal-regulated kinases (MEK/ERK) cascade. Calcineurin dephosphorylates nuclear factor of activated T cells (NFAT), allowing its nuclear translocation. PKC allows NF-κB nuclear translocation by removing of the inhibitor (IkB), while JNK and ERK activate AP1. In B cells, light grey, BCR/CD19/CD21 stimulation initiates a phosphorylation cascade starting with the activation of the kinases Lyn and SYK, leading to the activation of B cell linker protein (BLNK), B Cell adaptor molecule for phosphoinositide 3-Kinase (PI3K) (BCAP) and PLCg, ultimately resulting in PKC, JNK and ERK activation. Both TCR and BCR signaling potentiate mitochondrial respiration and activate metabolic pathways through their action on the complex formed by PI3K, protein kinase B (PKB/AKT) and mammalian target of rapamycin (mTOR). These signaling cascades are potentiated by ROS (H2O2 and O2-.) from NADPH oxidases (NOX/DUAOX) and from the mitochondria (red arrows). In some instances, NOX triggers the oxidative modification of ZAP70 and LCK to precipitate their degradation and blunt activation (dashed bloc red line).

The engagement of the B-cell receptor (BCR) or T-cell receptor (TCR) provides the specific signal 1, which in association with signal 2 coming from the co-costimulatory receptors, triggers intracellular phosphorylation cascades (**Figure 1**). This results in activation of the transcription factors activator protein 1 (AP1), nuclear factor (NF)-κB, nuclear factor of activated T cells (NFAT), Oct binding factor (OBF)-1/OCA-B (OCA-B/OBF-1 and Pip/interferon regulatory factor (IRF)-4, which are critical for T and B lymphocyte activation (**Figure 1**) (35, 41–45). Early research demonstrated that ROS scavengers such as N-acetyl cysteine (NAC) inhibit NF-κB activation following exposure to phorbol 12-myristate 13-acetate, tumor necrosis factor (TNF)-α, or interleukin-1 (IL-1), indicating that ROS are involved in physiological activation pathways (46, 47).

## ROS Contribute to TCR Signaling

Actually, within minutes of TCR stimulation, there is a production of both O^2-.^ and H_2_O_2_, which seems to originate from different TCR signaling pathways (48). Specifically, studies in Jurkat T cells showed that ROS increase the phosphorylation and activity of p56lck, ZAP-70, protein kinase C (PKC) and intracellular Ca^2+^ levels (**Figure 1**) (49–51). This results in a phosphoinositide-3 kinase (PI3K)/AKT/mTOR-, Myc- and ERRα-dependent augmentation of the global metabolism (52–56). It was further demonstrated that T cells from p47^phox^-deficient mice do not undergo TCR-induced H_2_O_2_ production, whereas TCR-induced O^2-.^ is unaffected following TCR stimulation in these cells, indicating that H_2_O_2_ originates from a lymphocyte-encoded NADPH oxidase (NOX) (57). Additionally, using superoxide scavenger treatments or superoxide deficiency in OT-II.Ncf1^m1J^ mice having CD4 T cell-specific superoxide deficiency, it was shown that superoxide is necessary for Th1 responses as well as IL-12R and proinflammatory chemokine ligand expression in CD4 T cells (58, 59). In fact, in T cells, ROS contribute not only to proximal but also to distal signaling pathways and modulate the activities of transcription factors NFAT, AP-1, and NF-kB to induce gene expression (60, 61). Activated T cells take up large amounts of glucose and produce lactate, indicating that they are primarily glycolytic (61, 62). Interestingly, during CD4 T-cell stimulation, mitochondrial oxygen consumption increases as an indication that mitochondrial function is also important for T-cell activation not only to support the glutamine requirement of these cells but also as a source of ROS (**Figure 1**) (61, 63). It was even shown that mitochondrial ROS specifically from respiratory chain complex III are required for CD4+ and CD8+ T cell expansion *in vivo* (61). Deletion of the Rieske iron sulfur protein (RISP), a subunit of mitochondrial complex III in T cells, resulted in a lack of oxidative phosphorylation and complex III-dependent ROS production and no expression of IL-2 upon CD3/CD28 stimulation (61). This phenotype was rescued by the addition of exogenous H_2_O_2_, clearly demonstrating the ROS requirement for full activation of the CD4 T cells (61). In this context, mitochondrial ROS were downstream of the TCR-mediated cytosolic and mitochondrial calcium increase, in agreement with the calcium dependency of mitochondrial TCA cycle dehydrogenases that fuel the electron transport chain (ETC) to increase mitochondrial membrane potential and ROS production (64, 65). Interestingly, mitochondrial ROS are also necessary for CD8 T-cell activation, as inhibition of the respiratory chain complex I decreases the production of H_2_O_2_, calcium flux, and ERK1/2 phosphorylation and impairs CD8 T-cell activation and proliferation (66). Complex I inhibition not only decreases activation of naive cells but also decreases interferon (IFN)-γ and TNF-α production as well as degranulation of effector and memory CD8+ T cells isolated from lymphocytic choriomeningitis virus-infected mice (66).

It is worth noting that some studies suggest mitochondria more than NADPH oxidase are the essential source of ROS involved in the activation process (61–66). This apparent discrepancy could come from the activation status (naïve/primed) or developmental state (CD8/CD4, T helper 1/2/9/17 or regulatory CD4 T cells) considered, which might have different ROS requirements for full activation (67). Nevertheless, collectively these results showed that ROS play a role in the activation and maturation of both CD8 and CD4 T cells (57, 61, 66, 68, 69). Mechanistically, by inhibiting phosphatases, ROS might tilt the balance toward phosphorylation, ultimately potentiating the activation of kinase cascades and transcription factors such as NFAT, which is critical for IL-2 production (61).

Interesting, although they are required, ROS levels must be kept in check by the glutathione-dependent antioxidant machinery (70, 71). Indeed, preventing glutathione (GSH) production impairs T-cell activation, as the energy and anabolic demands of these cells can no longer be met (72). GSH deficiency alters mammalian target of rapamycin (mTOR) and Myc activation, preventing the metabolic switch to glycolysis and glutaminolysis in an adenosine monophosphate–activated protein kinase (AMPK)-dependent manner (62, 73). Paradoxically, it was reported that ROS can also downregulate T-cell activation by regulating the degradation of signaling molecules and the activation of cytoskeletal proteins (74, 75). To prevent excessive ROS from triggering the mitochondrial permeability transition pore (PTP) opening and causing cell death, CD4 T cells upregulate microRNA (miR)-23a, which targets peptidylprolyl isomerase F (PPIF or Cyclophilin D), a key regulator of the PTP. The reduction in PPIF is expected to keep the mitochondrial PTP closed and reduce the escape of ROS, preserving CD4+ T-cell survival during the early hypermetabolic and inflammatory state of the activation process (76).

## ROS Contribute to BCR Signaling

Unlike that of T cells, B-cell metabolism is less well characterized. However, it was recently demonstrated that energy demand is elevated during antigen (Ag)-driven proliferation and differentiation (77, 78). B-cell stimulation with lipopolysaccharide (LPS) or anti-immunoglobulin M (IgM) antibodies drastically increases glucose import, although increased mitochondrial respiration still occurs, suggesting that here again mitochondrial function is important (**Figure 1**) (79, 80). Interestingly, ROS production in response to BCR stimulation occurs in two waves. An early NADPH oxidase 2-dependent ROS increase take place within minutes of BCR stimulation, and a second wave of increasing ROS levels from the mitochondria occurs at later time point (81). B cells deficient in early Nox2-dependent ROS production have no defects in proximal BCR signaling, cell activation or the ability to mount an antibody response following T cell-dependent Ag stimulation (81). However, preventing the later ROS increase attenuates BCR-dependent signaling, leading to defective activation, proliferation and response to BCR stimulation (81). These results indicate that the continuous production of mitochondrial ROS at later times during the activation process is critical for BCR signaling and optimal Ag-induced B-cell activation and proliferation, in agreement with findings from gene set enrichment analysis showing upregulation of OXPHOS and the TCA cycle in activated B cells (79, 81).

Lymphocyte activation clearly requires a metabolic reprograming for a diversification of the source of energy and biosynthetic building blocks (62, 82, 83). Therefore, one could wonder whether the observed increase in ROS during cell activation could be a consequence of this metabolic reprograming and reciprocally any genetic manipulation of the ROS input could also alter the metabolism of these cells, questioning the real significance of the ROS in the activation process? This latter possibility is readily excluded by the fact that exogenous H_2_O_2_ can rescue lymphocyte activation in the context of genetic ablation of complex III (61, 66, 79, 81). Taken together, these results clearly demonstrate that cell intrinsic ROS signaling participates in the activation processes of both B and T lymphocytes.

## ROS and Lymphocyte Viability

**Figure 2:**
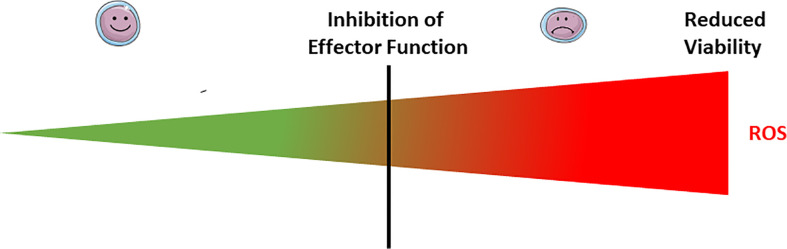
Exogenous ROS modulate lymphocyte effector functions and viability. At low doses of microenvironmental ROS, both B and T lymphocytes have normal effector function (smiley face lymphocytes). Exposure to mild doses of exogenous ROS affects the lymphocyte effector functions (weary face lymphocytes), while acute exposure to high doses affects their viability. The threshold between mild and acute exposure strongly depends upon the lymphocyte subset and maturation status.

We have seen that one of the proximal events following TCR signaling is an increase in ROS production both in the form of ${\text{O}}_{2}^{-}$ and H_2_O_2_. One direct consequence of the increase in these ROS in the context of T cell blasts is the initiation of activation-induced cell death (AICD) following the induction of FasL expression (84, 85). In fact, downstream of the TCR engagement, activated ZAP70 phosphorylates liker of activated T cells (LAT), which docks phospholipase Cγ1 that generates inositol 3 phosphate (IP3) and diacylglycerol (DAG) (84). DAG activates protein kinase C_θ_ (PKC_θ_) and its translocation into the mitochondria to enhance the production of ROS in a mitochondrial complex I-dependent manner, which is necessary for the expression of the ligand of the death receptor Fas (FasL) (84). FasL engages Fas receptor and triggers apoptotic cell death, a process where mitochondrial ROS further play a role, as it was later shown that caspase 3 can induced a ROS-dependent cell death by cleaving the respiratory chain complex I subunit NDUFS1 (30). We have also shown that ROS potentiate the apoptotic cascade by amplifying the release of apoptogenic factor from the mitochondria and increasing oligonucleosomal DNA fragmentation (86). Moreover, exposure to exogenous H_2_O_2_ differentially affects T-cell viability, according to their subset and maturation status. Central memory and effector memory T cells are more sensitive to H_2_O_2_ followed by naïve T cells, among which the CD8+ effector memory T-cell compartment is more sensitive to even low doses of H_2_O_2_ (**Figure 2**) (87, 88). In this context, exogenous H_2_O_2_ exposure triggers cell death in a mitochondrial pathway-dependent manner (87, 89). T cells treated with H_2_O_2_ experience the opening of the mitochondrial permeability transition pore (PTP), a rapid decrease in the mitochondrial transmembrane potential ΔΨm, and the release of cytochrome C (89). Blocking the mitochondrial PTP opening or interference with the respiratory electron transport chain with rotenone or menadione abrogated H_2_O_2_ cytotoxicity (89). Interestingly, antimycin A, a respiratory chain complex III inhibitor that increases the release of mitochondrial ROS, enhanced apoptosis, while overexpression of Bcl-2 and the viral anti-apoptotic proteins BHRF-1 and E1B 19K counteracted H_2_O_2_-induced T-cell apoptosis (89). Furthermore, inhibition of the transcription factor NF-κB protected cells from H_2_O_2-_induced cell death in a process that likely relies on the expression of a death effector gene such as p53 (89). Paradoxically, T regulatory cells, which have lower intracellular ROS levels, are particularly protected from H_2_O_2_-dependent inhibition of suppressive function and H_2_O_2_-induced death (90). Taken together, the higher sensitivity of effector memory CD8 T cells combined with the reduced susceptibility of T regulatory cells to H_2_O_2_-induced death suggest that the oxidized tumor microenvironment (TME) may be a particularly inhospitable site for CD8 T cells and detrimental to T cell-based adoptive cell transfer therapies. This is even more critical as effector memory T cells are the primary phenotype of cells administered during such therapeutic protocols. Thus, research is needed to determine the effect of the TME of chimeric antigen receptor (CAR)-T cell therapies. Beyond apoptosis, ROS critically regulate T-cell viability through the induction of ferroptosis. Both Ag-specific CD8+ and CD4+ T cells deficient for glutathione peroxidase 4 (Gpx4) are unable to expand or protect against viral and parasitic infection (91). This phenotype can be rescued by dietary vitamin E supplementation, indicating that lipid peroxidation-dependent ferroptosis plays a critical role in the T-cell depletion during these antigenic challenges (91).

Exposure of B cells to relatively massive doses (100–250 µM) of H_2_O_2_ has little effect on cell viability in the short term (30 minutes). However, 5 days later, half of the cells have died, even in the presence of CD40 stimulation (92). These doses of H_2_O_2_ although massive are still in the physiological range, as it was estimated that in the proximity of activated neutrophils and macrophages the H_2_O_2_ concentration can reach the 100s of µM (93–95). Moreover, this exogenous H_2_O_2_ exposure completely suppresses the ability to CD40 stimulation to trigger antibody production (**Figure 2**) (92). In fact, exogenous H_2_O_2_ exposure dissociates TRAF2 from CD40, leading to inefficient IKK phosphorylation, IκBα degradation, and NF-κB activation, which altogether severely compromises B-cell activation (92).

## ROS-Mediated Lymphocyte Dysfunction

As serial killers, cytotoxic T lymphocytes and NK cells must recognize, engage, kill, and detach from the first target cell before moving to the next (96–98). Detachment of the effector cell from its target requires its repolarization through the reorganization of its cytoskeleton for the disassembly of the first immunological synapse (99). Effector killer cells are quite sensitive to the redox status of their immediate environment (100–102). Oxidizing reagents curb killer cell degranulation, consequently inhibiting their cytotoxicity (102, 103). Interestingly, it was reported that oxidized low-density lipoprotein (ox-LDL), used as oxidizing agent, inhibits killer cell degranulation (102). Pretreatment of NK cells or co-incubation of NK/target cell conjugates with non-cytotoxic doses of ox-LDL markedly and significantly reduces the NK cytotoxic activity against U937 tumor cells (102). This reduced NK cell cytotoxicity is not the consequence of their inability to engage the target cells, because the number of NK:target cell conjugates was not affected nor were the expression levels of CD11a, CD11b, CD18, CD2, and CD62L, key adhesion molecules involved in the effector–target cell interaction (102). Mechanistically, ox-LDL triggers a partial depolarization of the microtubule network that is critical for the polarization of the cytotoxic granules toward the immunological synapse formed between the effector and target cells. Similarly, exposure of mitogen-stimulated peripheral blood mononuclear cells (PBMCs) to ox-LDL reduces their production of TNF-α, IFN-γ and IL-12 (102). Likewise, exogenous and endogenous nitric oxide (NO) inhibits degranulation of lymphokine activated killer (LAK) cells (103). NO inhibits LAK cell exocytosis in part by decreasing the expression of RAS, a critical component of the exocytic signaling cascade, following destabilization of RAS mRNA (103). NO acts by interfering with the mRNA-stabilizing factor HuR, which binds and stabilizes AU-rich elements of the mRNA 3’-untranslated region (104). It was further demonstrated that ROS induced oxidation of the C-terminal portion of the TCRζ chain, and the membrane proximal domain of p56(lck) and cofilin promote their degradation or inactivation, suggesting that ROS can also curb the TCR signaling cascade (74, 75). Similarly, increasing evidence suggests that the dynamics of the immunological synapse can be regulated by ROS through their direct or indirect effects *via* plasma membrane polarization on calcium signaling and effector cell cytoskeletal reorganization (99, 102, 105–107). As stated earlier, activation of the lymphocytes after engagement of their receptor initiates a phosphorylation cascade, resulting in, among other things, the mobilization of intracellular Ca^2+^ stores, which is essential for the gene expression crucial for lymphocyte activation and the development of adaptive immunity (99, 108–111). Depletion of Ca^2+^ stored in the endoplasmic reticulum triggers store-operated Ca^2+^ entry (SOCE). Compared to Orai1, the Ca^2+^ channel involved in SOCE, Orai3 lacks the redox-sensitive cysteine 195 and therefore is redox-insensitive. Co-expression of Orail3 with Orai1 reduces SOCE sensitivity to ROS inhibition. Consequently, it is not surprising that T lymphocytes display upregulated Orai3 expression during their differentiation into effector T cells. This means that the modulation of the Orai1:Orai3 ratio could be a possible mechanism by which effector T lymphocytes preserve some responsiveness in oxidized environments, such as the hypoxic TME or inflamed tissues (106, 112).

ROS also regulate the effector function of B cells. Overexpression of a phosphorylation-defective mutant of succinate dehydrogenase A to model excessive mitochondrial ROS production suppresses Ig production, germinal center (GC) formation, and GC B-cell proliferation following an encounter with T cell-dependent Ag. Excessive mitochondrial ROS production also suppresses Ig production against T cell-independent Ag (113) as well as BCR-dependent Lyn, Btk, and PLCγ2 phosphorylation and CD19 expression. From these collective results, it was hypothesized that excessive mitochondrial ROS dampen B-cell activation most likely by reducing CD19 expression (113). Overall, it seems that mild to moderate exposure to exogenous ROS affects the lymphocyte effector functions, while acute exposure affects their viability (**Figure 2**). The situation is complicated by the fact that the threshold between mild, moderate and acute exposure strongly depends upon the lymphocyte subset and maturation status.

## ROS in Immune Cell Dysfunction: The Case of Autoimmunity

As we have discussed earlier, endogenous ROS contribute to lymphocyte activation; however, depending of the lymphocyte activation and/or differentiation status, exogenous ROS can affect their effector function and viability. Thus, we also would like to discuss whether ROS could play a role in the pathogenesis of immune-related disorders such as autoimmunity where the wrath of the immune response mistargets self-antigens (autoantigens). For instance, abnormal functions of T helper (Th)-17 cells, which in a normal setting are essential to fight against extracellular bacteria (114–116), are involved in multiple chronic inflammatory disorders such as psoriasis, multiple sclerosis (MS), inflammatory bowel disease (IBD), Sjögren’s syndrome and rheumatoid arthritis (114). Interestingly, using sublethal doses of oligomycin A, an inhibitor of the respiratory chain ATP synthase/complex V, it was shown that mitochondrial oxidative phosphorylation (OXPHOS) plays a pivotal role in for Th17-mediated autoimmunity (117). Oligomycin treatment abolished Th17 pathogenicity by altering the expression of Th17 pathogenic signature genes, such as transforming growth factor beta 3 (TGFβ3), interleukin 23 receptor (IL-23R), signal transducer and activator of transcription 4 (Stat4), and G protein-coupled receptor 65 (Gpr65), while genes inversely associated with Th17 pathogenicity such as suppressor of cytokine signaling 3 (Socs3) and IL-10R subunit alpha (IL-10Ra) were upregulated (117). Although the authors did not directly test this possibility, it is very likely that mitochondrial ROS could be involved in this process, as in their experimental condition the oligomycin treatment severely suppresses the basal mitochondrial oxygen consumption rate, which could result in a severe reduction in mitochondrial ROS, indicating that ROS could protect against the pathogenicity of Th17 cells (117). This agrees with previously work by Tse and coworkers showing that prevention of ${\text{O}}_{2}^{-}$ production by macrophages and T cells skews T-cell polarization toward Th17 (118). Although they used a model of NOX-deficiency, collectively these results agree that the absence of ROS alters T-cell lineage commitment, pointing to a role for superoxide in the modulation Th17 *versus* Th1 T cell responses (118). From a more clinical stand point, it was shown that the hypomorphic allele of the Ncf1 gene encoding for p47^phox^, a subunit of NOX2, is one of the strongest genetic predispositions for autoimmune arthritis, autoimmune encephalomyelitis and systemic lupus erythematosus (SLE), which are associated with increased numbers of autoreactive T cells (119–122). Interestingly, results from two clinical trials, one using N acetyl cysteine the other Sirolimus to modulate respectively cellular GSH content and mTOR activity resulted in the improvement of SLE condition suggesting that modulation of the mitochondrial ROS output could also contribute to regulate pro inflammatory T cell development (123, 124). Furthermore, by downmodulating the efficacy of antigen processing, ROS may further contribute to limiting the activation of autoreactive lymphocytes. In this regard, in the early stage of the processes, ROS should not simply be considered as effectors to eliminate invading pathogens, but also as modulators to fine-tune the inflammatory response depending on the timing, the site and the level of their production (125, 126). By contrast, in the context of dysregulated and prolonged chronic inflammation, the local microenvironment is characterized by a low nutrient levels, increased lactate production, decreased pH, hypoxia and an increase level of ROS, which collectively lead to excessive tissue destruction. At this later stage, this excessive tissue destruction could promote the accessibility to cryptic neoantigens favoring the progression and exacerbation of autoimmunity (126).

## ROS in the Process of Cytotoxic Lymphocyte Killing

Cytotoxic lymphocytes are particularly efficient at eliminating target cancer cells and virally infected cells. They mainly used the cytotoxic granule pathway relying on the degranulation of the pore forming protein perforin and a family of five serine proteases call granzymes in human (127–129). Although the granzymes trigger very distinct cell death pathways, we found that granzyme A and B (GA and GB) share the ability to induce ROS-dependent cell death. It was demonstrated that GA induces ROS-dependent death that is independent of the mitochondrial outer membrane permeabilization (MOMP) and insensitive to BCL2 but has all the morphological features of apoptosis (127, 130–133). We also showed that ROS are necessary for the rapid cell death induction by GB. We found that K562 cells treated with a sublytic concentration of perforin (P) and GB undergo a rapid increase in ROS production and cell death that is inhibited in the presence of the well-characterized antioxidants N-acetyl cysteine (NAC), superoxide scavenger MnTBAP, or the mitochondrial targeted superoxide scavenger MitoQ (134). Moreover, GB and P-induced ROS and cell death are completely absent in pseudo rho cells deficient for mitochondrial DNA (mtDNA) and therefore lacking a functional respiratory chain (134). Both GA and GB induce ROS release from isolated intact mitochondria in the absence of cytoplasmic fraction S100 (130). Using organelle proteomics and bioinformatics, we found that GA and GB cleave NDUFS3, NDUFV1, NDUFS1 and NDUFS2 iron-sulfur (Fe-S) cluster-containing subunits of the respiratory chain complex I (86, 130, 132, 135). Cleavage of complex I subunits exposes iron sulfur clusters and dramatically increases electron leak from the respiratory chain, leading to a rapid and sustained mitocentric ROS production, loss of complex I, II, and III activities, disorganization of the respiratory chain, mitochondrial respiration impairment, and loss of mitochondrial cristae junctions (86, 130, 132, 135, 136). It is worth noting that another study has also suggested the contribution of NOX as source of ROS during GB-mediated cell death (137). However, we found that, GB-mediated killing of mouse embryonic fibroblasts (MEFs) from NOX-deficient animals proceeds as in wild-type MEFs (86). GB induction of mitocentric ROS promotes apoptogenic factor release and oligonucleosomal DNA fragmentation (138, 139). Although granzymes do not express a mitochondrial targeting signal, they enter the mitochondria independently from the TOM40 complex, the organelle entry gate, and use instead the SAM50 channel (136, 140). SAM50 is the core channel of the mitochondrial sorting and assembly machinery dedicated to the insertion of *de novo* β-barrel proteins into the mitochondrial outer membrane (141–144). Preventing the entry of granzymes into the target cell mitochondria alters their cytotoxicity. Using a model of human glioma, a very aggressive primary brain tumor for which there is no cure, we showed that granzyme mitochondrial entry is also essential for the reduction of tumor burden *in vivo* (136, 140). Collectively, these interesting results also indicated that respiratory chain complex I is at the crosstalk of GA, GB and caspase 3, three different cell death pathways. Complex I targeting is also conserved across phylum from bacteria to mammals. In collaboration with the Walch's group we showed GA- and GB-mediated disruption of bacterial complex I is also a necessary step for bacterial death (145). The central role of complex I alteration during cell death suggests that it is a very important step whose full range of function has yet to be unraveled. For more about the antimicrobial action of the granzymes, we refer readers to the review of the oxidative and non-oxidative antimicrobial activities of the granzymes by Marilyne Lavergne on this same research topic.

## Cancer, Oxidative Stress, and Cytotoxic Lymphocytes

**Figure 3:**
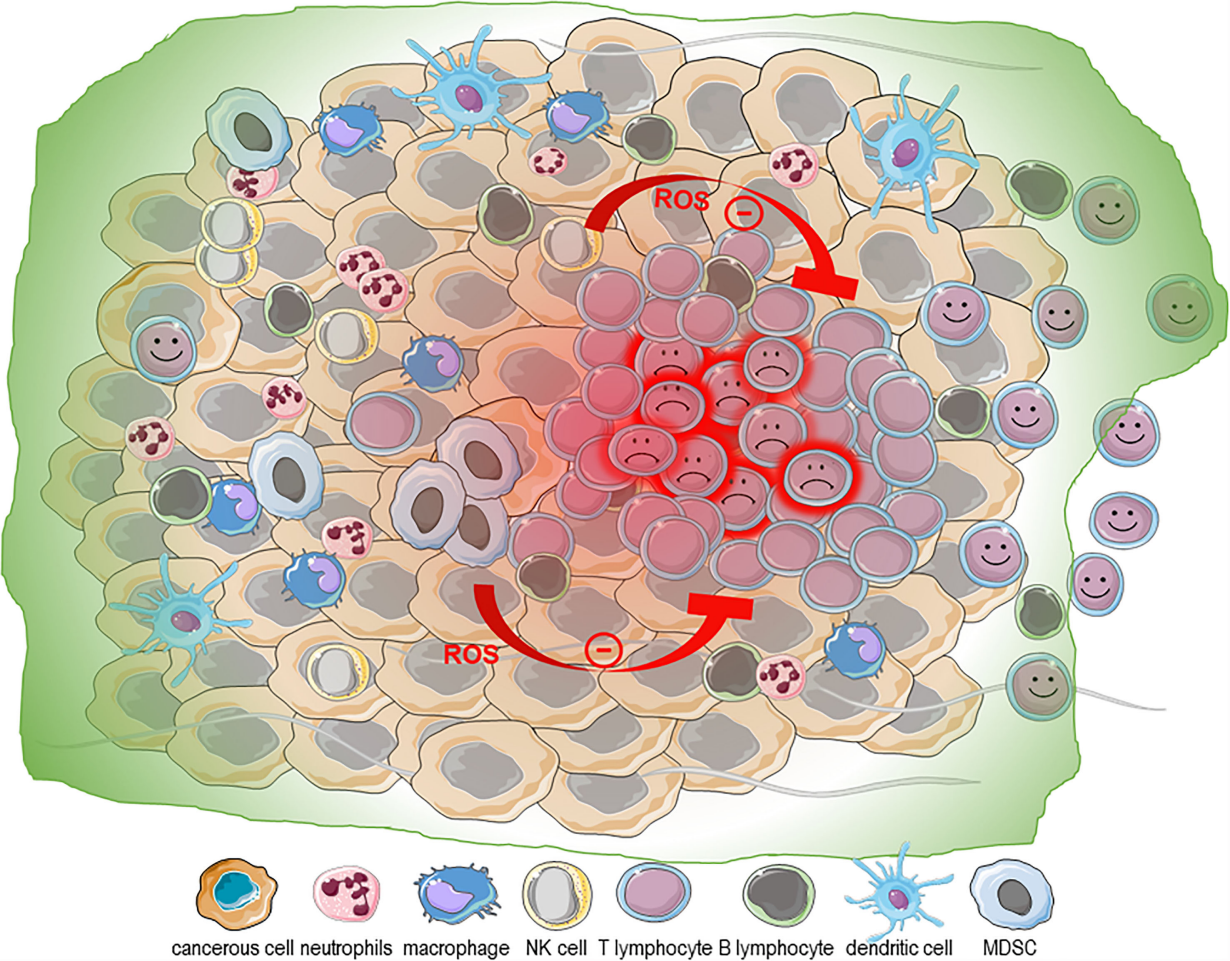
ROS in the tumor microenvironment. The metabolic stress imposed by the neoplastic transformation and uncontrolled proliferation combined with the hypoxic nature and marked production of ROS contribute to the chronic inflammation state of the tumor microenvironment (TME). The oxidative nature of the TME is exacerbated by its infiltration with immune cells with altered phenotypes [neutrophils, macrophages and myeloid derived suppressor cells [MDSCs)]. NK cells and CD8+ effector memory T cells are particularly sensitive to even low doses of H_2_O_2_, while the T regulatory cells are protected from H_2_O_2_-dependent inhibition of their suppressive function and H_2_O_2_-induced death. Taken together, this supports that the oxidized TME may be a particularly inhospitable site for NK cells and CD8 T cells and detrimental to T cell-based adoptive cell transfer therapies.

Uncontrolled proliferation and neoplastic transformation come with enormous demands for energy and macromolecule building blocks. These demands impose a severe metabolic stress, requiring a striking reprogramming of the cancer cell metabolism (146, 147). The resulting altered metabolism combined with the hypoxic nature of the TME is accompanied by marked production of ROS (148–151). This overproduction of ROS activates the cellular antioxidant response based on enzymatic and non-enzymatic antioxidant molecules, which is under the transcriptional control of the transcription factor nuclear factor (erythroid-derived 2)-like 2 (NRF2). Three isoforms of superoxide dismutase (SOD), cytosolic CuZn-SOD (SOD1), mitochondrial Mn-SOD (SOD2), and extracellular EC-SOD (SOD3), are involved in the rapid dismutation of O^2-^ into H_2_O_2_ (15, 16). The homotetrameric catalase converts H_2_O_2_ into water using NADPH as a cofactor (15, 17). The glutathione peroxidases (GPx) use glutathione (GSH) and reduce H_2_O_2_ and lipid hydroperoxides (15, 18). H_2_O_2_ removal also involves thioredoxin (TRX), thioredoxin reductase (TRR), thioredoxin peroxidase (PRX) and glutaredoxins (15). The most abundant non-enzymatic antioxidant molecule in the cell is GSH, which participates in the reduction of H_2_O_2_ into H_2_O and O_2_, and is thereby oxidized to form GSSG. GSSG is then recycled into GSH by glutathione reductase still using as electron donor NAD(P)H. GSH also maintains aqueous and lipophilic levels of the antioxidant ascorbic acid (vitamin C) and α-tocopherol (vitamin E), respectively. Nevertheless, when this antioxidant system is overwhelmed, the pro-oxidant/anti-oxidant equilibrium is lost, and a state of oxidative stress is reached where the cells accumulate oxidative damage in all type of macromolecules, including DNA, RNA, lipids and proteins, which could lead to cell death (152–154). Oxidative DNA modifications generate 8-hydroxy-2′-deoxyguanosine, which contributes to the accumulation of mutations that enhance aging and carcinogenesis (155). Consequently, transformed cells adapt and reach new redox balance, and paradoxically, ROS instead of killing, stimulate tumor development and progression by promoting cell proliferation through their mitogenic action as activator of extracellular-regulated kinase 1/2 (ERK1/2). This induces ligand-independent receptor tyrosine kinase (RTK) activation, activating Src kinase, NF-κB and phosphatidylinositol-3 kinase (PI3K)/Akt, to enable evasion of apoptosis and anoikis as well as to induce metalloproteinase (MMP) release in the extracellular matrix to favor invasion and promote angiogenesis (156–161). ROS also contribute to epithelial to mesenchymal transition (EMT), an important process in the metastatic dissemination of cancer cells (2). Importantly, in the TME, cancer cells reprogram other cells, such as cancer-associated fibroblasts (CAFs), endothelial cells and cancer-associated macrophages (CAMs), in a ROS-dependent manner to favor tumor progression. CAFs contribute to tumor growth by promoting the tumor angiogenesis by secreting VEGF and angiopoietin, by generating anti-apoptotic factors and by the secretion of chemokines (CCL2 and CCL5) and MMPs to promote the dissemination while blocking the immune response through the secretion of immunosuppressive cytokines IL-6, IL-10 and TGF-β (162–164). This marked production of ROS also alters the phenotype of innate immune cells infiltrating the tumor parenchyma, contributing to the noxious nature of the TME (165–168). Interestingly, it is worth noting that a direct link exists between the environmental ROS of the TME and inflammation (169). Intracellular ROS may regulate EMT in a NF-κB– and hypoxia-inducible factor 1 (HIF-1α)–dependent manner in a process requiring the activity of cyclooxygenase-2 (COX-2), the first enzyme in the synthesis of prostaglandins, prostacyclin and thromboxanes including prostaglandin E2 (PGE2). This suggests that this oxidized microenvironment favors a state of chronic inflammation in the TME. As stated earlier cytotoxic lymphocytes (NK cells and cytotoxic T lymphocytes) play an essential role in the immune response against cancer (97, 170–177). It is therefore not surprising that harnessing the power of these innate and adaptive cytotoxic immune cells during immune check point blockage (ICB) or CAR-T/NK cell immunotherapies has produced very encouraging results (178–180). As we have discussed earlier, NK cells and CD8+ effector memory T cells are particularly sensitive to even low doses of H_2_O_2_ while the T regulatory cells are protected from H_2_O_2_-dependent inhibition of their suppressive function and H_2_O_2_-induced death (87, 88, 90). Accordingly, tumor-infiltrating lymphocytes must adapt to this oxidized microenvironment among other things by modulating the ratio ORAI1:ORAI3 expression (106, 112). Despite these adaptation mechanisms, as we have seen earlier, exposure to exogenous ROS can severely dampen lymphocytes’ effector function, making the TME particularly hostile to infiltrating lymphocytes (181). Interestingly and counter intuitively, the inflamed nature of the TME further contributes to making the TME hostile for lymphocytes and NK cells. Indeed, it was recently reported that tumor-derived PGE2 achieves immune evasion by inhibiting NK cell-mediated remodeling of the TME and unleashing of cytotoxic T cells (173). Interestingly, F2-isoprostanes (F2-IsoPs) and isolevuglandins (IsoLGs), which are oxidized derivatives of PGE2, are extremely relevant disease biomarkers, as they are directly involved in the pathological processes (induction of inflammatory pathways, modulation of immune response, and induction of cell death) (182–184). Since inflammation is closely linked to ROS production, whether the oxidized form of PGE2 contributes to this immune evasion needs to be investigated. Collectively, the evidence supports that the oxidized nature of the TME is likely to affect the efficiency of infiltrating anti-tumor lymphocytes and the development of strategies to enable lymphocytes to withstand the oxidized nature of the TME could improve immunotherapies (**Figure 3**).

## Concluding Remarks

Based on the available evidence, both NOX- and mitochondrial-derived ROS play critical roles in lymphocyte activation, development, effector function, cytotoxicity, viability but also dysfunction. To this regard ROS directly contribute to both physiological and pathological adaptive immune responses. ROS from both sources contribute to the activation process of lymphocytes, however, based on strong genetic evidence relying on hypomorphic allele of the Ncf1 gene encoding for p47^phox^, it is tempting to suggest that NOX-derived ROS would have a preponderant role as modulators to fine-tune the inflammatory response depending on the timing, the site and the level of their production. But more investigations are still required to seal this case. Moreover, since ROS also participate in innate cell function by potentiating the killing ability of phagocytes, an essential step in the antigen processing and presentation function of these phagocytes, ROS also indirectly contribute to adaptive immunity though the interplay between innate and adaptive immunity. Further characterization of the complex functions of ROS in lymphocyte biology will bring new insight for understanding the pathological conditions in which lymphocyte function is either detrimental or beneficial.

## Author Contributions

DM, EB, and MW wrote and made the illustrations. All authors contributed to the article and approved the submitted version.

## Funding

This work was supported by grants from UNIPD SID 2018 and ERC starting grant ERC-2010-StG_20091118.

## Conflict of Interest

The authors declare that the research was conducted in the absence of any commercial or financial relationships that could be construed as a potential conflict of interest.

## Publisher’s Note

All claims expressed in this article are solely those of the authors and do not necessarily represent those of their affiliated organizations, or those of the publisher, the editors and the reviewers. Any product that may be evaluated in this article, or claim that may be made by its manufacturer, is not guaranteed or endorsed by the publisher.

## References

[1] Weidinger A Kozlov AV. Biological Activities of Reactive Oxygen and Nitrogen Species: Oxidative Stress *Versus* Signal Transduction. Biomolecules (2015) 5:472–84. doi: 10.3390/biom5020472 PMC4496681 25884116

[2] Omori E Inagaki M Mishina Y Matsumoto K Ninomiya-Tsuji J. Epithelial Transforming Growth Factor Beta-Activated Kinase 1 (TAK1) is Activated Through Two Independent Mechanisms and Regulates Reactive Oxygen Species. Proc Natl Acad Sci USA (2012) 109:3365–70. doi: 10.1073/pnas.1116188109 PMC3295251 22331902

[3] Murphy MP. How Mitochondria Produce Reactive Oxygen Species. Biochem J (2009) 417:1–13. doi: 10.1042/BJ20081386 19061483 PMC2605959

[4] Cruz CM Rinna A Forman HJ Ventura AL Persechini PM Ojcius DM. ATP Activates a Reactive Oxygen Species-Dependent Oxidative Stress Response and Secretion of Proinflammatory Cytokines in Macrophages. J Biol Chem (2007) 282:2871–9. doi: 10.1074/jbc.M608083200 PMC2693903 17132626

[5] Dostert C Petrilli V Van Bruggen R Steele C Mossman BT Tschopp J. Innate Immune Activation Through Nalp3 Inflammasome Sensing of Asbestos and Silica. Science (2008) 320:674–7. doi: 10.1126/science.1156995 PMC2396588 18403674

[6] Schroder K Tschopp J. The Inflammasomes. Cell (2010) 140:821–32. doi: 10.1016/j.cell.2010.01.040 20303873

[7] Winterbourn CC. Reconciling the Chemistry and Biology of Reactive Oxygen Species. Nat Chem Biol (2008) 4:278–86. doi: 10.1038/nchembio.85 18421291

[8] Bashan N Kovsan J Kachko I Ovadia H Rudich A. Positive and Negative Regulation of Insulin Signaling by Reactive Oxygen and Nitrogen Species. Physiol Rev (2009) 89:27–71. doi: 10.1152/physrev.00014.2008 19126754

[9] Cash TP Pan Y Simon MC. Reactive Oxygen Species and Cellular Oxygen Sensing. Free Radical Biol Med (2007) 43:1219–25. doi: 10.1016/j.freeradbiomed.2007.07.001 PMC2696222 17893032

[10] Klimova T Chandel NS. Mitochondrial Complex III Regulates Hypoxic Activation of HIF. Cell Death Differ (2008) 15:660–6. doi: 10.1038/sj.cdd.4402307 18219320

[11] Ullevig S Zhao Q Lee CF Seok Kim H Zamora D Asmis R. NADPH Oxidase 4 Mediates Monocyte Priming and Accelerated Chemotaxis Induced by Metabolic Stress. Arterioscler Thromb Vasc Biol (2012) 32:415–26. doi: 10.1161/ATVBAHA.111.238899 PMC3262086 22095986

[12] Crosas-Molist E Bertran E Sancho P Lopez-Luque J Fernando J Sanchez A. The NADPH Oxidase NOX4 Inhibits Hepatocyte Proliferation and Liver Cancer Progression. Free Radical Biol Med (2014) 69:338–47. doi: 10.1016/j.freeradbiomed.2014.01.040 24509161

[13] Wang Y Zang QS Liu Z Wu Q Maass D Dulan G. Regulation of VEGF-Induced Endothelial Cell Migration by Mitochondrial Reactive Oxygen Species. Am J Physiol Cell Physiol (2011) 301:C695–704. doi: 10.1152/ajpcell.00322.2010 PMC3174570 21653897

[14] Lennicke C Rahn J Lichtenfels R Wessjohann LA Seliger B. Hydrogen Peroxide - Production, Fate and Role in Redox Signaling of Tumor Cells. Cell Commun Signal (2015) 13:39. doi: 10.1186/s12964-015-0118-6 26369938 PMC4570748

[15] Birben E Sahiner UM Sackesen C Erzurum S Kalayci O. Oxidative Stress and Antioxidant Defense. World Allergy Organ J (2012) 5:9–19. doi: 10.1097/WOX.0b013e3182439613 23268465 PMC3488923

[16] Zelko IN Mariani TJ Folz RJ. Superoxide Dismutase Multigene Family: A Comparison of the CuZn-SOD (SOD1), Mn-SOD (SOD2), and EC-SOD (SOD3) Gene Structures, Evolution, and Expression. Free Radical Biol Med (2002) 33:337–49. doi: 10.1016/S0891-5849(02)00905-X 12126755

[17] Kirkman HN Rolfo M Ferraris AM Gaetani GF. Mechanisms of Protection of Catalase by NADPH. Kinetics and Stoichiometry. J Biol Chem (1999) 274:13908–14. doi: 10.1074/jbc.274.20.13908 10318800

[18] Brigelius-Flohe R Maiorino M. Glutathione Peroxidases. Biochim Biophys Acta (2013) 1830:3289–303. doi: 10.1016/j.bbagen.2012.11.020 23201771

[19] Cadet J Wagner JR. DNA Base Damage by Reactive Oxygen Species, Oxidizing Agents, and UV Radiation. Cold Spring Harb Perspect Biol (2013) 5:1–18. doi: 10.1101/cshperspect.a012559 PMC3552502 23378590

[20] Cadet J Wagner JR. Oxidatively Generated Base Damage to Cellular DNA by Hydroxyl Radical and One-Electron Oxidants: Similarities and Differences. Arch Biochem biophysics (2014) 557:47–54. doi: 10.1016/j.abb.2014.05.001 24820329

[21] Panasenko OM Evgina SA Driomina ES Sharov VS Sergienko VI Vladimirov YA. Hypochlorite Induces Lipid Peroxidation in Blood Lipoproteins and Phospholipid Liposomes. Free Radical Biol Med (1995) 19:133–40. doi: 10.1016/0891-5849(94)00211-2 7649485

[22] Salgo MG Squadrito GL Pryor WA. Peroxynitrite Causes Apoptosis in Rat Thymocytes. Biochem Biophys Res Commun (1995) 215:1111–8. doi: 10.1006/bbrc.1995.2578 7488038

[23] Salgo MG Stone K Squadrito GL Battista JR Pryor WA. Peroxynitrite Causes DNA Nicks in Plasmid Pbr322. Biochem Biophys Res Commun (1995) 210:1025–30. doi: 10.1006/bbrc.1995.1759 7763229

[24] Koppenol WH. The Centennial of the Fenton Reaction. Free Radical Biol Med (1993) 15:645–51. doi: 10.1016/0891-5849(93)90168-T 8138191

[25] Fulda S Gorman AM Hori O Samali A. Cellular Stress Responses: Cell Survival and Cell Death. Int J Cell Biol (2010) 2010:214074. doi: 10.1155/2010/214074 20182529 PMC2825543

[26] Schenk B Fulda S. Reactive Oxygen Species Regulate Smac Mimetic/TNFalpha-Induced Necroptotic Signaling and Cell Death. Oncogene (2015) 34:5796–806. doi: 10.1038/onc.2015.35 25867066

[27] Fulda S. Regulation of Necroptosis Signaling and Cell Death by Reactive Oxygen Species. Biol Chem (2016) 397:657–60. doi: 10.1515/hsz-2016-0102 26918269

[28] Cecconi F Levine B. The Role of Autophagy in Mammalian Development: Cell Makeover Rather Than Cell Death. Dev Cell (2008) 15:344–57. doi: 10.1016/j.devcel.2008.08.012 PMC2688784 18804433

[29] Green DR Kroemer G. The Pathophysiology of Mitochondrial Cell Death. Science (2004) 305:626–9. doi: 10.1126/science.1099320 15286356

[30] Ricci JE Munoz-Pinedo C Fitzgerald P Bailly-Maitre B Perkins GA Yadava N. Disruption of Mitochondrial Function During Apoptosis Is Mediated by Caspase Cleavage of the P75 Subunit of Complex I of the Electron Transport Chain. Cell (2004) 117:773–86. doi: 10.1016/j.cell.2004.05.008 15186778

[31] Tait SW Green DR. Mitochondria and Cell Death: Outer Membrane Permeabilization and Beyond. Nat Rev Mol Cell Biol (2010) 11:621–32. doi: 10.1038/nrm2952 20683470

[32] Dixon SJ Lemberg KM Lamprecht MR Skouta R Zaitsev EM Gleason CE. Ferroptosis: An Iron-Dependent Form of Nonapoptotic Cell Death. Cell (2012) 149:1060–72. doi: 10.1016/j.cell.2012.03.042 PMC3367386 22632970

[33] Chaplin DD. Overview of the Immune Response. J Allergy Clin Immunol (2010) 125:S3–23. doi: 10.1016/j.jaci.2009.12.980 20176265 PMC2923430

[34] Turvey SE Broide DH. Innate Immunity. J Allergy Clin Immunol (2010) 125:S24–32. doi: 10.1016/j.jaci.2009.07.016 PMC2832725 19932920

[35] Bonilla FA Oettgen HC. Adaptive Immunity. J Allergy Clin Immunol (2010) 125:S33–40. doi: 10.1016/j.jaci.2009.09.017 20061006

[36] Atarashi K Tanoue T Shima T Imaoka A Kuwahara T Momose Y. Induction of Colonic Regulatory T Cells by Indigenous Clostridium Species. Science (2011) 331:337–41. doi: 10.1126/science.1198469 PMC3969237 21205640

[37] Lee YK Mazmanian SK. Has the Microbiota Played a Critical Role in the Evolution of the Adaptive Immune System? Science (2010) 330:1768–73. doi: 10.1126/science.1195568 PMC3159383 21205662

[38] Bianchi ME. DAMPs, PAMPs and Alarmins: All We Need to Know About Danger. J Leukoc Biol (2007) 81:1–5. doi: 10.1189/jlb.0306164 17032697

[39] Alder MN Rogozin IB Iyer LM Glazko GV Cooper MD Pancer Z. Diversity and Function of Adaptive Immune Receptors in a Jawless Vertebrate. Science (2005) 310:1970–3. doi: 10.1126/science.1119420 16373579

[40] Wardemann H Yurasov S Schaefer A Young JW Meffre E Nussenzweig MC. Predominant Autoantibody Production by Early Human B Cell Precursors. Science (2003) 301:1374–7. doi: 10.1126/science.1086907 12920303

[41] Kim U Qin XF Gong S Stevens S Luo Y Nussenzweig M. The B-Cell-Specific Transcription Coactivator OCA-B/OBF-1/Bob-1 Is Essential for Normal Production of Immunoglobulin Isotypes. Nature (1996) 383:542–7. doi: 10.1038/383542a0 8849728

[42] Huppa JB Gleimer M Sumen C Davis MM. Continuous T Cell Receptor Signaling Required for Synapse Maintenance and Full Effector Potential. Nat Immunol (2003) 4:749–55. doi: 10.1038/ni951 12858171

[43] Chen L Widhopf G Huynh L Rassenti L Rai KR Weiss A. Expression of ZAP-70 Is Associated With Increased B-Cell Receptor Signaling in Chronic Lymphocytic Leukemia. Blood (2002) 100:4609–14. doi: 10.1182/blood-2002-06-1683 12393534

[44] Schubart DB Rolink A Kosco-Vilbois MH Botteri F Matthias P. B-Cell-Specific Coactivator OBF-1/OCA-B/Bob1 Required for Immune Response and Germinal Centre Formation. Nature (1996) 383:538–42. doi: 10.1038/383538a0 8849727

[45] Brass AL Zhu AQ Singh H. Assembly Requirements of PU.1-Pip (IRF-4) Activator Complexes: Inhibiting Function *In Vivo* Using Fused Dimers. EMBO J (1999) 18:977–91. doi: 10.1093/emboj/18.4.977 PMC1171190 10022840

[46] Schreck R Rieber P Baeuerle PA. Reactive Oxygen Intermediates as Apparently Widely Used Messengers in the Activation of the NF-Kappa B Transcription Factor and HIV-1. EMBO J (1991) 10:2247–58. doi: 10.1002/j.1460-2075.1991.tb07761.x PMC452914 2065663

[47] Staal FJ Roederer M Herzenberg LA Herzenberg LA. Intracellular Thiols Regulate Activation of Nuclear Factor Kappa B and Transcription of Human Immunodeficiency Virus. Proc Natl Acad Sci United States America (1990) 87:9943–7. doi: 10.1073/pnas.87.24.9943 PMC55290 2263644

[48] Devadas S Zaritskaya L Rhee SG Oberley L Williams MS. Discrete Generation of Superoxide and Hydrogen Peroxide by T Cell Receptor Stimulation: Selective Regulation of Mitogen-Activated Protein Kinase Activation and Fas Ligand Expression. J Exp Med (2002) 195:59–70. doi: 10.1084/jem.20010659 11781366 PMC2196010

[49] Hardwick JS Sefton BM. Activation of the Lck Tyrosine Protein Kinase by Hydrogen Peroxide Requires the Phosphorylation of Tyr-394. Proc Natl Acad Sci USA (1995) 92:4527–31. doi: 10.1073/pnas.92.10.4527 PMC41977 7538674

[50] Schieven GL Mittler RS Nadler SG Kirihara JM Bolen JB Kanner SB. ZAP-70 Tyrosine Kinase, CD45, and T Cell Receptor Involvement in UV- and H2O2-Induced T Cell Signal Transduction. J Biol Chem (1994) 269:20718–26. doi: 10.1016/S0021-9258(17)32051-3 8051172

[51] Cemerski S Cantagrel A Van Meerwijk JP Romagnoli P. Reactive Oxygen Species Differentially Affect T Cell Receptor-Signaling Pathways. J Biol Chem (2002) 277:19585–93. doi: 10.1074/jbc.M111451200 11916964

[52] MacIver NJ Michalek RD Rathmell JC. Metabolic Regulation of T Lymphocytes. Annu Rev Immunol (2013) 31:259–83. doi: 10.1146/annurev-immunol-032712-095956 PMC3606674 23298210

[53] Powell JD Pollizzi KN Heikamp EB Horton MR. Regulation of Immune Responses by mTOR. Annu Rev Immunol (2012) 30:39–68. doi: 10.1146/annurev-immunol-020711-075024 22136167 PMC3616892

[54] Frauwirth KA Riley JL Harris MH Parry RV Rathmell JC Plas DR. The CD28 Signaling Pathway Regulates Glucose Metabolism. Immunity (2002) 16:769–77. doi: 10.1016/S1074-7613(02)00323-0 12121659

[55] Michalek RD Gerriets VA Nichols AG Inoue M Kazmin D Chang CY. Estrogen-Related Receptor-Alpha is a Metabolic Regulator of Effector T-Cell Activation and Differentiation. Proc Natl Acad Sci USA (2011) 108:18348–53. doi: 10.1073/pnas.1108856108 PMC3215012 22042850

[56] Wang R Dillon CP Shi LZ Milasta S Carter R Finkelstein D. The Transcription Factor Myc Controls Metabolic Reprogramming Upon T Lymphocyte Activation. Immunity (2011) 35:871–82. doi: 10.1016/j.immuni.2011.09.021 PMC3248798 22195744

[57] Jackson SH Devadas S Kwon J Pinto LA Williams MS. T Cells Express a Phagocyte-Type NADPH Oxidase That Is Activated After T Cell Receptor Stimulation. Nat Immunol (2004) 5:818–27. doi: 10.1038/ni1096 15258578

[58] Padgett LE Tse HM. NADPH Oxidase-Derived Superoxide Provides a Third Signal for CD4 T Cell Effector Responses. J Immunol (2016) 197:1733–42. doi: 10.4049/jimmunol.1502581 PMC4992601 27474077

[59] Tse HM Milton MJ Schreiner S Profozich JL Trucco M Piganelli JD. Disruption of Innate-Mediated Proinflammatory Cytokine and Reactive Oxygen Species Third Signal Leads to Antigen-Specific Hyporesponsiveness. J Immunol (2007) 178:908–17. doi: 10.4049/jimmunol.178.2.908 17202352

[60] Kaminski MM Sauer SW Kaminski M Opp S Ruppert T Grigaravicius P. T Cell Activation is Driven by an ADP-Dependent Glucokinase Linking Enhanced Glycolysis With Mitochondrial Reactive Oxygen Species Generation. Cell Rep (2012) 2:1300–15. doi: 10.1016/j.celrep.2012.10.009 23168256

[61] Sena LA Li S Jairaman A Prakriya M Ezponda T Hildeman DA. Mitochondria are Required for Antigen-Specific T Cell Activation Through Reactive Oxygen Species Signaling. Immunity (2013) 38:225–36. doi: 10.1016/j.immuni.2012.10.020 PMC3582741 23415911

[62] Geltink RIK Kyle RL Pearce EL. Unraveling the Complex Interplay Between T Cell Metabolism and Function. Annu Rev Immunol (2018) 36:461–88. doi: 10.1146/annurev-immunol-042617-053019 PMC6323527 29677474

[63] DeBerardinis RJ Mancuso A Daikhin E Nissim I Yudkoff M Wehrli S. Beyond Aerobic Glycolysis: Transformed Cells can Engage in Glutamine Metabolism That Exceeds the Requirement for Protein and Nucleotide Synthesis. Proc Natl Acad Sci USA (2007) 104:19345–50. doi: 10.1073/pnas.0709747104 PMC2148292 18032601

[64] Denton RM. Regulation of Mitochondrial Dehydrogenases by Calcium Ions. Biochim Biophys Acta (2009) 1787:1309–16. doi: 10.1016/j.bbabio.2009.01.005 19413950

[65] McCormack JG Denton RM. Role of Calcium Ions in the Regulation of Intramitochondrial Metabolism. Properties of the Ca2+-Sensitive Dehydrogenases Within Intact Uncoupled Mitochondria From the White and Brown Adipose Tissue of the Rat. Biochem J (1980) 190:95–105. doi: 10.1042/bj1900095 6778477 PMC1162067

[66] Yi JS Holbrook BC Michalek RD Laniewski NG Grayson JM. Electron Transport Complex I Is Required for CD8+ T Cell Function. J Immunol (2006) 177:852–62. doi: 10.4049/jimmunol.177.2.852 16818739

[67] Peng HY Lucavs J Ballard D Das JK Kumar A Wang L. Metabolic Reprogramming and Reactive Oxygen Species in T Cell Immunity. Front Immunol (2021) 12:652687. doi: 10.3389/fimmu.2021.652687 33868291 PMC8044852

[68] Gulow K Kaminski M Darvas K Suss D Li-Weber M Krammer PH. HIV-1 Trans-Activator of Transcription Substitutes for Oxidative Signaling in Activation-Induced T Cell Death. J Immunol (2005) 174:5249–60. doi: 10.4049/jimmunol.174.9.5249 15843521

[69] Kwon J Shatynski KE Chen H Morand S de Deken X Miot F. The Nonphagocytic NADPH Oxidase Duox1 Mediates a Positive Feedback Loop During T Cell Receptor Signaling Science Signaling. Science Signaling (2010) 3:ra59. doi: 10.1126/scisignal.2000976 PMC2941205 20682913

[70] Mak TW Grusdat M Duncan GS Dostert C Nonnenmacher Y Cox M. Glutathione Primes T Cell Metabolism for Inflammation. Immunity (2017) 46:1089–90. doi: 10.1016/j.immuni.2017.06.009 28636957

[71] Muri J Kopf M. Redox Regulation of Immunometabolism. Nat Rev Immunol (2021) 21:363–81. doi: 10.1038/s41577-020-00478-8 33340021

[72] Mak TW Grusdat M Duncan GS Dostert C Nonnenmacher Y Cox M. Glutathione Primes T Cell Metabolism for Inflammation. Immunity (2017) 46:675–89. doi: 10.1016/j.immuni.2017.03.019 28423341

[73] Tamas P Hawley SA Clarke RG Mustard KJ Green K Hardie DG. Regulation of the Energy Sensor AMP-Activated Protein Kinase by Antigen Receptor and Ca2+ in T Lymphocytes. J Exp Med (2006) 203:1665–70. doi: 10.1084/jem.20052469 PMC2118355 16818670

[74] Cemerski S van Meerwijk JP Romagnoli P. Oxidative-Stress-Induced T Lymphocyte Hyporesponsiveness Is Caused by Structural Modification Rather Than Proteasomal Degradation of Crucial TCR Signaling Molecules. Eur J Immunol (2003) 33:2178–85. doi: 10.1002/eji.200323898 12884292

[75] Samstag Y John I Wabnitz GH. Cofilin: A Redox Sensitive Mediator of Actin Dynamics During T-Cell Activation and Migration. Immunol Rev (2013) 256:30–47. doi: 10.1111/imr.12115 24117811 PMC3884758

[76] Zhang B Liu SQ Li C Lykken E Jiang S Wong E. MicroRNA-23a Curbs Necrosis During Early T Cell Activation by Enforcing Intracellular Reactive Oxygen Species Equilibrium. Immunity (2016) 44:568–81. doi: 10.1016/j.immuni.2016.01.007 PMC4794397 26921109

[77] Boothby M Rickert RC. Metabolic Regulation of the Immune Humoral Response. Immunity (2017) 46:743–55. doi: 10.1016/j.immuni.2017.04.009 PMC5640164 28514675

[78] Aronov M Tirosh B. Metabolic Control of Plasma Cell Differentiation- What We Know and What We Don't Know. J Clin Immunol (2016) 36 Suppl 1:12–7. doi: 10.1007/s10875-016-0246-9 26910101

[79] Waters LR Ahsan FM Wolf DM Shirihai O Teitell MA. Initial B Cell Activation Induces Metabolic Reprogramming and Mitochondrial Remodeling. iScience (2018) 5:99–109. doi: 10.1016/j.isci.2018.07.005 30240649 PMC6123864

[80] Caro-Maldonado A Wang R Nichols AG Kuraoka M Milasta S Sun LD. Metabolic Reprogramming is Required for Antibody Production That is Suppressed in Anergic But Exaggerated in Chronically BAFF-Exposed B Cells. J Immunol (2014) 192:3626–36. doi: 10.4049/jimmunol.1302062 PMC3984038 24616478

[81] Wheeler ML Defranco AL. Prolonged Production of Reactive Oxygen Species in Response to B Cell Receptor Stimulation Promotes B Cell Activation and Proliferation. J Immunol (2012) 189:4405–16. doi: 10.4049/jimmunol.1201433 PMC3515638 23024271

[82] O'Neill LA Kishton RJ Rathmell J. A Guide to Immunometabolism for Immunologists. Nat Rev Immunol (2016) 16:553–65. doi: 10.1038/nri.2016.70 PMC5001910 27396447

[83] Makowski L Chaib M Rathmell JC. Immunometabolism: From Basic Mechanisms to Translation. Immunol Rev (2020) 295:5–14. doi: 10.1111/imr.12858 32320073 PMC8056251

[84] Kaminski M Kiessling M Suss D Krammer PH Gulow K. Novel Role for Mitochondria: Protein Kinase Ctheta-Dependent Oxidative Signaling Organelles in Activation-Induced T-Cell Death. Mol Cell Biol (2007) 27:3625–39. doi: 10.1128/MCB.02295-06 PMC1900004 17339328

[85] Li-Weber M Weigand MA Giaisi M Suss D Treiber MK Baumann S. Vitamin E Inhibits CD95 Ligand Expression and Protects T Cells From Activation-Induced Cell Death. J Clin Invest (2002) 110:681–90. doi: 10.1172/JCI0215073 PMC151103 12208869

[86] Jacquemin G Margiotta D Kasahara A Bassoy EY Walch M Thiery J. Granzyme B-Induced Mitochondrial ROS are Required for Apoptosis. Cell Death Differ (2015) 22:862–74. doi: 10.1038/cdd.2014.180 PMC4392081 25361078

[87] Takahashi A Hanson MG Norell HR Havelka AM Kono K Malmberg KJ. Preferential Cell Death of CD8+ Effector Memory (CCR7-CD45RA-) T Cells by Hydrogen Peroxide-Induced Oxidative Stress. J Immunol (2005) 174:6080–7. doi: 10.4049/jimmunol.174.10.6080 15879102

[88] Belikov AV Schraven B Simeoni L. T Cells and Reactive Oxygen Species. J BioMed Sci (2015) 22:85. doi: 10.1186/s12929-015-0194-3 26471060 PMC4608155

[89] Dumont A Hehner SP Hofmann TG Ueffing M Droge W Schmitz ML. Hydrogen Peroxide-Induced Apoptosis Is CD95-Independent, Requires the Release of Mitochondria-Derived Reactive Oxygen Species and the Activation of NF-Kappab. Oncogene (1999) 18:747–57. doi: 10.1038/sj.onc.1202325 9989825

[90] Mougiakakos D Johansson CC Kiessling R. Naturally Occurring Regulatory T Cells Show Reduced Sensitivity Toward Oxidative Stress-Induced Cell Death. Blood (2009) 113:3542–5. doi: 10.1182/blood-2008-09-181040 19050306

[91] Matsushita M Freigang S Schneider C Conrad M Bornkamm GW Kopf M. T Cell Lipid Peroxidation Induces Ferroptosis and Prevents Immunity to Infection. J Exp Med (2015) 212:555–68. doi: 10.1084/jem.20140857 PMC4387287 25824823

[92] Liu J Yoshida Y Yamashita U. Suppressive Effect of Reactive Oxygen Species on CD40-Induced B Cell Activation. FEBS Lett (2007) 581:5043–9. doi: 10.1016/j.febslet.2007.09.042 17919601

[93] Nathan CF Root RK. Hydrogen Peroxide Release From Mouse Peritoneal Macrophages: Dependence on Sequential Activation and Triggering. J Exp Med (1977) 146:1648–62. doi: 10.1084/jem.146.6.1648 PMC2181906 925614

[94] Nathan CF. Neutrophil Activation on Biological Surfaces. Massive Secretion of Hydrogen Peroxide in Response to Products of Macrophages and Lymphocytes. J Clin Invest (1987) 80:1550–60. doi: 10.1172/JCI113241 PMC442423 2445780

[95] Nathan CF. Secretory Products of Macrophages. J Clin Invest (1987) 79:319–26. doi: 10.1172/JCI112815 PMC424063 3543052

[96] Choi PJ Mitchison TJ. Imaging Burst Kinetics and Spatial Coordination During Serial Killing by Single Natural Killer Cells. Proc Natl Acad Sci USA (2013) 110:6488–93. doi: 10.1073/pnas.1221312110 PMC3631668 23576740

[97] Rothstein TL Mage M Jones G McHugh LL. Cytotoxic T Lymphocyte Sequential Killing of Immobilized Allogeneic Tumor Target Cells Measured by Time-Lapse Microcinematography. J Immunol (1978) 121:1652–6. 309477

[98] Sanderson CJ. The Mechanism of T-Cell Mediated Cytotoxicity. VIII. Zeiosis Corresponds to Irreversible Phase (Programming for Lysis) in Steps Leading to Lysis. Immunology (1981) 42:201–6. PMC1458082 6970176

[99] Kummerow C Junker C Kruse K Rieger H Quintana A Hoth M. The Immunological Synapse Controls Local and Global Calcium Signals in T Lymphocytes. Immunol Rev (2009) 231:132–47. doi: 10.1111/j.1600-065X.2009.00811.x 19754894

[100] Hoffmann PR Berry MJ. The Influence of Selenium on Immune Responses. Mol Nutr Food Res (2008) 52:1273–80. doi: 10.1002/mnfr.200700330 PMC3723386 18384097

[101] Li X Yin D Yin J Chen Q Wang R. Dietary Selenium Protect Against Redox-Mediated Immune Suppression Induced by Methylmercury Exposure. Food Chem Toxicol an Int J published Br Ind Biol Res Assoc (2014) 72:169–77. doi: 10.1016/j.fct.2014.07.023 25057806

[102] Malorni W Straface E Di Genova G Fattorossi A Rivabene R Camponeschi B. Oxidized Low-Density Lipoproteins Affect Natural Killer Cell Activity by Impairing Cytoskeleton Function and Altering the Cytokine Network. Exp Cell Res (1997) 236:436–45. doi: 10.1006/excr.1997.3736 9367628

[103] Ferlito M Irani K Faraday N Lowenstein CJ. Nitric Oxide Inhibits Exocytosis of Cytolytic Granules From Lymphokine-Activated Killer Cells. Proc Natl Acad Sci USA (2006) 103:11689–94. doi: 10.1073/pnas.0600275103 PMC1544231 16857739

[104] Akool el S Kleinert H Hamada FM Abdelwahab MH Forstermann U Pfeilschifter J. Nitric Oxide Increases the Decay of Matrix Metalloproteinase 9 mRNA by Inhibiting the Expression of mRNA-Stabilizing Factor HuR. Mol Cell Biol (2003) 23:4901–16. doi: 10.1128/MCB.23.14.4901-4916.2003 PMC162218 12832476

[105] Goitre L Pergolizzi B Ferro E Trabalzini L Retta SF. Molecular Crosstalk Between Integrins and Cadherins: Do Reactive Oxygen Species Set the Talk? J Signal transduction (2012) 2012:807682. doi: 10.1155/2012/807682 PMC3238397 22203898

[106] Nunes P Demaurex N. Redox Regulation of Store-Operated Ca2+ Entry. Antioxidants Redox Signaling (2014) 21:915–32. doi: 10.1089/ars.2013.5615 PMC4116092 24053140

[107] Nimnual AS Taylor LJ Bar-Sagi D. Redox-Dependent Downregulation of Rho by Rac. Nat Cell Biol (2003) 5:236–41. doi: 10.1038/ncb938 12598902

[108] Feske S Gwack Y Prakriya M Srikanth S Puppel SH Tanasa B. A Mutation in Orai1 Causes Immune Deficiency by Abrogating CRAC Channel Function. Nature (2006) 441:179–85. doi: 10.1038/nature04702 16582901

[109] Hogan PG Chen L Nardone J Rao A. Transcriptional Regulation by Calcium, Calcineurin, and NFAT. Genes Dev (2003) 17:2205–32. doi: 10.1101/gad.1102703 12975316

[110] Yeromin AV Zhang SL Jiang W Yu Y Safrina O Cahalan MD. Molecular Identification of the CRAC Channel by Altered Ion Selectivity in a Mutant of Orai. Nature (2006) 443:226–9. doi: 10.1038/nature05108 PMC2756048 16921385

[111] Zhang SL Yu Y Roos J Kozak JA Deerinck TJ Ellisman MH. STIM1 Is a Ca2+ Sensor That Activates CRAC Channels and Migrates From the Ca2+ Store to the Plasma Membrane. Nature (2005) 437:902–5. doi: 10.1038/nature04147 PMC1618826 16208375

[112] Bogeski I Kummerow C Al-Ansary D Schwarz EC Koehler R Kozai D. Differential Redox Regulation of ORAI Ion Channels: A Mechanism to Tune Cellular Calcium Signaling. Sci Signaling (2010) 3:ra24. doi: 10.1126/scisignal.2000672 20354224

[113] Ogura M Inoue T Yamaki J Homma MK Kurosaki T Homma Y. Mitochondrial Reactive Oxygen Species Suppress Humoral Immune Response Through Reduction of CD19 Expression in B Cells in Mice. Eur J Immunol (2017) 47:406–18. doi: 10.1002/eji.201646342 27883180

[114] Patel DD Kuchroo VK. Th17 Cell Pathway in Human Immunity: Lessons From Genetics and Therapeutic Interventions. Immunity (2015) 43:1040–51. doi: 10.1016/j.immuni.2015.12.003 26682981

[115] Jutel M Agache I Bonini S Burks AW Calderon M Canonica W. International Consensus on Allergen Immunotherapy II: Mechanisms, Standardization, and Pharmacoeconomics. J Allergy Clin Immunol (2016) 137:358–68. doi: 10.1016/j.jaci.2015.12.1300 26853128

[116] Annunziato F Romagnani C Romagnani S. The 3 Major Types of Innate and Adaptive Cell-Mediated Effector Immunity. J Allergy Clin Immunol (2015) 135:626–35. doi: 10.1016/j.jaci.2014.11.001 25528359

[117] Shin B Benavides GA Geng J Koralov SB Hu H Darley-Usmar VM. Mitochondrial Oxidative Phosphorylation Regulates the Fate Decision Between Pathogenic Th17 and Regulatory T Cells. Cell Rep (2020) 30:1898–909.e4. doi: 10.1016/j.celrep.2020.01.022 32049019 PMC9059282

[118] Tse HM Thayer TC Steele C Cuda CM Morel L Piganelli JD. NADPH Oxidase Deficiency Regulates Th Lineage Commitment and Modulates Autoimmunity. J Immunol (2010) 185:5247–58. doi: 10.4049/jimmunol.1001472 PMC3190397 20881184

[119] Olofsson P Holmberg J Tordsson J Lu S Akerstrom B Holmdahl R. Positional Identification of Ncf1 as a Gene That Regulates Arthritis Severity in Rats. Nat Genet (2003) 33:25–32. doi: 10.1038/ng1058 12461526

[120] Hultqvist M Olofsson P Holmberg J Backstrom BT Tordsson J Holmdahl R. Enhanced Autoimmunity, Arthritis, and Encephalomyelitis in Mice With a Reduced Oxidative Burst Due to a Mutation in the Ncf1 Gene. Proc Natl Acad Sci United States America (2004) 101:12646–51. doi: 10.1073/pnas.0403831101 PMC515111 15310853

[121] Campbell AM Kashgarian M Shlomchik MJ. NADPH Oxidase Inhibits the Pathogenesis of Systemic Lupus Erythematosus. Sci Transl Med (2012) 4:157ra41. doi: 10.1126/scitranslmed.3004801 PMC3704198 23100627

[122] Kienhofer D Hahn J Stoof J Csepregi JZ Reinwald C Urbonaviciute V. Experimental Lupus is Aggravated in Mouse Strains With Impaired Induction of Neutrophil Extracellular Traps. JCI Insight (2017) 2:1–13. doi: 10.1172/jci.insight.92920 PMC5436535 28515366

[123] Lai ZW Hanczko R Bonilla E Caza TN Clair B Bartos A. N-Acetylcysteine Reduces Disease Activity by Blocking Mammalian Target of Rapamycin in T Cells From Systemic Lupus Erythematosus Patients: A Randomized, Double-Blind, Placebo-Controlled Trial. Arthritis Rheum (2012) 64:2937–46. doi: 10.1002/art.34502 PMC3411859 22549432

[124] Lai ZW Kelly R Winans T Marchena I Shadakshari A Yu J. Sirolimus in Patients With Clinically Active Systemic Lupus Erythematosus Resistant to, or Intolerant of, Conventional Medications: A Single-Arm, Open-Label, Phase 1/2 Trial. Lancet (2018) 391:1186–96. doi: 10.1016/S0140-6736(18)30485-9 PMC5891154 29551338

[125] Hultqvist M Olsson LM Gelderman KA Holmdahl R. The Protective Role of ROS in Autoimmune Disease. Trends Immunol (2009) 30:201–8. doi: 10.1016/j.it.2009.03.004 19356981

[126] Hoffmann MH Griffiths HR. The Dual Role of Reactive Oxygen Species in Autoimmune and Inflammatory Diseases: Evidence From Preclinical Models. Free Radical Biol Med (2018) 125:62–71. doi: 10.1016/j.freeradbiomed.2018.03.016 29550327

[127] Chowdhury D Lieberman J. Death by a Thousand Cuts: Granzyme Pathways of Programmed Cell Death. Annu Rev Immunol (2008) 26:389–420. doi: 10.1146/annurev.immunol.26.021607.090404 18304003 PMC2790083

[128] Trapani JA Smyth MJ. Functional Significance of the Perforin/Granzyme Cell Death Pathway. Nat Rev Immunol (2002) 2:735–47. doi: 10.1038/nri911 12360212

[129] Trapani JA. Granzymes, Cytotoxic Granules and Cell Death: The Early Work of Dr. Jurg Tschopp. Cell Death Differ (2012) 19:21–7. doi: 10.1038/cdd.2011.156 PMC3252834 22095283

[130] Martinvalet D Zhu P Lieberman J. Granzyme A Induces Caspase-Independent Mitochondrial Damage, a Required First Step for Apoptosis. Immunity (2005) 22:355–70. doi: 10.1016/j.immuni.2005.02.004 15780992

[131] Chowdhury D Beresford PJ Zhu P Zhang D Sung JS Demple B. The Exonuclease TREX1 is in the SET Complex and Acts in Concert With NM23-H1 to Degrade DNA During Granzyme A-Mediated Cell Death. Mol Cell (2006) 23:133–42. doi: 10.1016/j.molcel.2006.06.005 16818237

[132] Martinvalet D Dykxhoorn DM Ferrini R Lieberman J. Granzyme A Cleaves a Mitochondrial Complex I Protein to Initiate Caspase-Independent Cell Death. Cell (2008) 133:681–92. doi: 10.1016/j.cell.2008.03.032 PMC2840390 18485875

[133] Beresford PJ Xia Z Greenberg AH Lieberman J. Granzyme A Loading Induces Rapid Cytolysis and a Novel Form of DNA Damage Independently of Caspase Activation. Immunity (1999) 10:585–94. doi: 10.1016/S1074-7613(00)80058-8 10367904

[134] Jacquemin G Margiotta D Kasahara A Bassoy EY Walch M Thiery J. Granzyme B-Induced Mitochondrial ROS Are Required for Apoptosis. Cell Death Differ (2015) 22:862–74. doi: 10.1038/cdd.2014.180 PMC4392081 25361078

[135] Martinvalet D. ROS Signaling During Granzyme B-Mediated Apoptosis. Mol Cell Oncol (2015) 2:e992639. doi: 10.4161/23723556.2014.992639 27308474 PMC4905311

[136] Chiusolo V Jacquemin G Yonca Bassoy E Vinet L Liguori L Walch M. Granzyme B Enters the Mitochondria in a Sam50-, Tim22- and Mthsp70-Dependent Manner to Induce Apoptosis. Cell Death Differ (2017) 24:747–58. doi: 10.1038/cdd.2017.3 PMC5384031 28338658

[137] Aguilo JI Anel A Catalan E Sebastian A Acin-Perez R Naval J. Granzyme B of Cytotoxic T Cells Induces Extramitochondrial Reactive Oxygen Species Production *via* Caspase-Dependent NADPH Oxidase Activation. Immunol Cell Biol (2010) 88:545–54. doi: 10.1038/icb.2010.5 20125115

[138] Shresta S MacIvor DM Heusel JW Russell JH Ley TJ. Natural Killer and Lymphokine-Activated Killer Cells Require Granzyme B for the Rapid Induction of Apoptosis in Susceptible Target Cells. Proc Natl Acad Sci USA (1995) 92:5679–83. doi: 10.1073/pnas.92.12.5679 PMC41760 7777569

[139] Parrish J Li L Klotz K Ledwich D Wang X Xue D. Mitochondrial Endonuclease G is Important for Apoptosis in C. Elegans. Nature (2001) 412:90–4. doi: 10.1038/35083608 11452313

[140] Lionello S Marzaro G Martinvalet D. SAM50, a Side Door to the Mitochondria: The Case of Cytotoxic Proteases. Pharmacol Res (2020) 160:105196. doi: 10.1016/j.phrs.2020.105196 32919042

[141] Kozjak-Pavlovic V Ross K Benlasfer N Kimmig S Karlas A Rudel T. Conserved Roles of Sam50 and Metaxins in VDAC Biogenesis. EMBO Rep (2007) 8:576–82. doi: 10.1038/sj.embor.7400982 PMC2002532 17510655

[142] Kutik S Stojanovski D Becker L Becker T Meinecke M Kruger V. Dissecting Membrane Insertion of Mitochondrial Beta-Barrel Proteins. Cell (2008) 132:1011–24. doi: 10.1016/j.cell.2008.01.028 18358813

[143] Noinaj N Kuszak AJ Gumbart JC Lukacik P Chang H Easley NC. Structural Insight Into the Biogenesis of Beta-Barrel Membrane Proteins. Nature (2013) 501:385–90. doi: 10.1038/nature12521 PMC3779476 23995689

[144] Paschen SA Waizenegger T Stan T Preuss M Cyrklaff M Hell K. Evolutionary Conservation of Biogenesis of Beta-Barrel Membrane Proteins. Nature (2003) 426:862–874. doi: 10.1038/nature02208 14685243

[145] Walch M Dotiwala F Mulik S Thiery J Kirchhausen T Clayberger C. Cytotoxic Cells Kill Intracellular Bacteria Through Granulysin-Mediated Delivery of Granzymes. Cell (2014) 157:1309–23. doi: 10.1016/j.cell.2014.03.062 PMC4090916 24906149

[146] Seyfried TN Flores R Poff AM D'Agostino DP Mukherjee P. Metabolic Therapy: A New Paradigm for Managing Malignant Brain Cancer. Cancer Lett (2015) 356:289–300. doi: 10.1016/j.canlet.2014.07.015 25069036

[147] Warburg O. On the Origin of Cancer Cells. Science (1956) 123:309–14. doi: 10.1126/science.123.3191.309 13298683

[148] Guzy RD Hoyos B Robin E Chen H Liu L Mansfield KD. Mitochondrial Complex III is Required for Hypoxia-Induced ROS Production and Cellular Oxygen Sensing. Cell Metab (2005) 1:401–8. doi: 10.1016/j.cmet.2005.05.001 16054089

[149] Chandel NS Maltepe E Goldwasser E Mathieu CE Simon MC Schumacker PT. Mitochondrial Reactive Oxygen Species Trigger Hypoxia-Induced Transcription. Proc Natl Acad Sci USA (1998) 95:11715–20. doi: 10.1073/pnas.95.20.11715 PMC21706 9751731

[150] Duranteau J Chandel NS Kulisz A Shao Z Schumacker PT. Intracellular Signaling by Reactive Oxygen Species During Hypoxia in Cardiomyocytes. J Biol Chem (1998) 273:11619–24. doi: 10.1074/jbc.273.19.11619 9565580

[151] Chandel NS McClintock DS Feliciano CE Wood TM Melendez JA Rodriguez AM. Reactive Oxygen Species Generated at Mitochondrial Complex III Stabilize Hypoxia-Inducible Factor-1alpha During Hypoxia: A Mechanism of O2 Sensing. J Biol Chem (2000) 275:25130–8. doi: 10.1074/jbc.M001914200 10833514

[152] Veskoukis AS Tsatsakis AM Kouretas D. Dietary Oxidative Stress and Antioxidant Defense With an Emphasis on Plant Extract Administration. Cell Stress Chaperones (2012) 17:11–21. doi: 10.1007/s12192-011-0293-3 21956695 PMC3227848

[153] Halliwell B Chirico S. Lipid Peroxidation: Its Mechanism, Measurement, and Significance. Am J Clin Nutr (1993) 57:715S–24S; discussion 24S-25S. doi: 10.1093/ajcn/57.5.715S 8475889

[154] Levine RL. Carbonyl Modified Proteins in Cellular Regulation, Aging, and Disease. Free Radical Biol Med (2002) 32:790–6. doi: 10.1016/S0891-5849(02)00765-7 11978480

[155] Matsui A Ikeda T Enomoto K Hosoda K Nakashima H Omae K. Increased Formation of Oxidative DNA Damage, 8-Hydroxy-2'-Deoxyguanosine, in Human Breast Cancer Tissue and Its Relationship to GSTP1 and COMT Genotypes. Cancer Lett (2000) 151:87–95. doi: 10.1016/S0304-3835(99)00424-3 10766427

[156] Matsuzawa A Ichijo H. Redox Control of Cell Fate by MAP Kinase: Physiological Roles of ASK1-MAP Kinase Pathway in Stress Signaling. Biochim Biophys Acta (2008) 1780:1325–36. doi: 10.1016/j.bbagen.2007.12.011 18206122

[157] Nguyen T Nioi P Pickett CB. The Nrf2-Antioxidant Response Element Signaling Pathway and Its Activation by Oxidative Stress. J Biol Chem (2009) 284:13291–5. doi: 10.1074/jbc.R900010200 PMC2679427 19182219

[158] Fei J Hong A Dobbins TA Jones D Lee CS Loo C. Prognostic Significance of Vascular Endothelial Growth Factor in Squamous Cell Carcinomas of the Tonsil in Relation to Human Papillomavirus Status and Epidermal Growth Factor Receptor. Ann Surg Oncol (2009) 16:2908–17. doi: 10.1245/s10434-009-0579-1 19603236

[159] Shi YH Wang YX Bingle L Gong LH Heng WJ Li Y. *In Vitro* Study of HIF-1 Activation and VEGF Release by bFGF in the T47D Breast Cancer Cell Line Under Normoxic Conditions: Involvement of PI-3k/Akt and MEK1/ERK Pathways. J Pathol (2005) 205:530–6. doi: 10.1002/path.1734 15714461

[160] Radisky DC Levy DD Littlepage LE Liu H Nelson CM Fata JE. Rac1b and Reactive Oxygen Species Mediate MMP-3-Induced EMT and Genomic Instability. Nature (2005) 436:123–7. doi: 10.1038/nature03688 PMC2784913 16001073

[161] Rhyu DY Yang Y Ha H Lee GT Song JS Uh ST. Role of Reactive Oxygen Species in TGF-Beta1-Induced Mitogen-Activated Protein Kinase Activation and Epithelial-Mesenchymal Transition in Renal Tubular Epithelial Cells. J Am Soc Nephrol JASN (2005) 16:667–75. doi: 10.1681/ASN.2004050425 15677311

[162] Kayamori K Sakamoto K Nakashima T Takayanagi H Morita K Omura K. Roles of Interleukin-6 and Parathyroid Hormone-Related Peptide in Osteoclast Formation Associated With Oral Cancers: Significance of Interleukin-6 Synthesized by Stromal Cells in Response to Cancer Cells. Am J Pathol (2010) 176:968–80. doi: 10.2353/ajpath.2010.090299 PMC2808100 20035059

[163] Tsuyada A Chow A Wu J Somlo G Chu P Loera S. CCL2 Mediates Cross-Talk Between Cancer Cells and Stromal Fibroblasts That Regulates Breast Cancer Stem Cells. Cancer Res (2012) 72:2768–79. doi: 10.1158/0008-5472.CAN-11-3567 PMC3367125 22472119

[164] Sotgia F Del Galdo F Casimiro MC Bonuccelli G Mercier I Whitaker-Menezes D. Caveolin-1-/- Null Mammary Stromal Fibroblasts Share Characteristics With Human Breast Cancer-Associated Fibroblasts. Am J Pathol (2009) 174:746–61. doi: 10.2353/ajpath.2009.080658 PMC2665737 19234134

[165] Masucci MT Minopoli M Carriero MV. Tumor Associated Neutrophils. Their Role in Tumorigenesis, Metastasis, Prognosis and Therapy. Front Oncol (2019) 9:1146. doi: 10.3389/fonc.2019.01146 31799175 PMC6874146

[166] Vincent J Mignot G Chalmin F Ladoire S Bruchard M Chevriaux A. 5-Fluorouracil Selectively Kills Tumor-Associated Myeloid-Derived Suppressor Cells Resulting in Enhanced T Cell-Dependent Antitumor Immunity. Cancer Res (2010) 70:3052–61. doi: 10.1158/0008-5472.CAN-09-3690 20388795

[167] Noy R Pollard JW. Tumor-Associated Macrophages: From Mechanisms to Therapy. Immunity (2014) 41:49–61. doi: 10.1016/j.immuni.2014.06.010 25035953 PMC4137410

[168] Liu T Han C Wang S Fang P Ma Z Xu L. Cancer-Associated Fibroblasts: An Emerging Target of Anti-Cancer Immunotherapy. J Hematol Oncol (2019) 12:86. doi: 10.1186/s13045-019-0770-1 31462327 PMC6714445

[169] Eltzschig HK Carmeliet P. Hypoxia and Inflammation. N Engl J Med (2011) 364:656–65. doi: 10.1056/NEJMra0910283 PMC3930928 21323543

[170] Jansen CS Prokhnevska N Master VA Sanda MG Carlisle JW Bilen MA. An Intra-Tumoral Niche Maintains and Differentiates Stem-Like CD8 T Cells. Nature (2019) 576:465–70. doi: 10.1038/s41586-019-1836-5 PMC7108171 31827286

[171] Barrow AD Edeling MA Trifonov V Luo J Goyal P Bohl B. Natural Killer Cells Control Tumor Growth by Sensing a Growth Factor. Cell (2018) 172:534–48.e19. doi: 10.1016/j.cell.2017.11.037 29275861 PMC6684025

[172] Guillerey C Huntington ND Smyth MJ. Targeting Natural Killer Cells in Cancer Immunotherapy. Nat Immunol (2016) 17:1025–36. doi: 10.1038/ni.3518 27540992

[173] Bonavita E Bromley CP Jonsson G Pelly VS Sahoo S Walwyn-Brown K. Antagonistic Inflammatory Phenotypes Dictate Tumor Fate and Response to Immune Checkpoint Blockade. Immunity (2020) 53:1215–29.e8. doi: 10.1016/j.immuni.2020.10.020 33220234 PMC7772804

[174] Rosenberg SA. Progress in Human Tumour Immunology and Immunotherapy. Nature (2001) 411:380–4. doi: 10.1038/35077246 11357146

[175] Herberman RB. Immunogenicity of Tumor Antigens. Biochim Biophys Acta (1977) 473:93–119. doi: 10.1016/0304-419X(77)90002-6 73384

[176] Greenberg PD. Adoptive T Cell Therapy of Tumors: Mechanisms Operative in the Recognition and Elimination of Tumor Cells. Adv Immunol (1991) 49:281–355. doi: 10.1016/S0065-2776(08)60778-6 1853786

[177] Golstein P Griffiths GM. An Early History of T Cell-Mediated Cytotoxicity. Nat Rev Immunol (2018) 18:527–35. doi: 10.1038/s41577-018-0009-3 29662120

[178] Sharma P Allison JP. Dissecting the Mechanisms of Immune Checkpoint Therapy. Nat Rev Immunol (2020) 20:75–6. doi: 10.1038/s41577-020-0275-8 31925406

[179] Wei SC Anang NAS Sharma R Andrews MC Reuben A Levine JH. Combination Anti-CTLA-4 Plus Anti-PD-1 Checkpoint Blockade Utilizes Cellular Mechanisms Partially Distinct From Monotherapies. Proc Natl Acad Sci USA (2019) 116:22699–709. doi: 10.1073/pnas.1821218116 PMC6842624 31636208

[180] Topalian SL Drake CG Pardoll DM. Immune Checkpoint Blockade: A Common Denominator Approach to Cancer Therapy. Cancer Cell (2015) 27:450–61. doi: 10.1016/j.ccell.2015.03.001 PMC4400238 25858804

[181] Chen X Song M Zhang B Zhang Y. Reactive Oxygen Species Regulate T Cell Immune Response in the Tumor Microenvironment. Oxid Med Cell Longev (2016) 2016:1580967. doi: 10.1155/2016/1580967 27547291 PMC4980531

[182] Frijhoff J Winyard PG Zarkovic N Davies SS Stocker R Cheng D. Clinical Relevance of Biomarkers of Oxidative Stress. Antioxidants Redox Signaling (2015) 23:1144–70. doi: 10.1089/ars.2015.6317 PMC4657513 26415143

[183] Brame CJ Boutaud O Davies SS Yang T Oates JA Roden D. Modification of Proteins by Isoketal-Containing Oxidized Phospholipids. J Biol Chem (2004) 279:13447–51. doi: 10.1074/jbc.M313349200 14715668

[184] Davies SS Amarnath V Montine KS Bernoud-Hubac N Boutaud O Montine TJ. Effects of Reactive Gamma-Ketoaldehydes Formed by the Isoprostane Pathway (Isoketals) and Cyclooxygenase Pathway (Levuglandins) on Proteasome Function. FASEB J (2002) 16:715–7. doi: 10.1096/fj.01-0696fje 11978738

